# Stratifying the risk of re-detachment: variables associated with outcome of vitrectomy for rhegmatogenous retinal detachment in a large UK cohort study

**DOI:** 10.1038/s41433-023-02388-0

**Published:** 2023-04-25

**Authors:** David Yorston, Paul H. J. Donachie, D. A. Laidlaw, David H. Steel, G. W. Aylward, Tom H. Williamson, the BEAVRS database study group, David Steel, Andrew Morris, Craig Goldsmith, Stephen Winder, Richard Sheard, Jonathan Smith, Tony Casswell, Diego Sanchez-Chicharro, Atiq Babar, Tim Cochrane, Vaughan Tanner, Vasileios Papastavrou, Deepak Vayalambrone, Tsveta Ivanova, Jonathan Park, Assad Jalil, Kurt Spiteri Cornish, Abdallah Ellabban, Sonali Tarafdar, Imran Khan, Edward Hughes, Kam Balaggan, Laura Wakely, Steve Charles, Huw Jenkins, Izabela Mitrut

**Affiliations:** 1 Gartnavel Hospital, Glasgow, G12 0YN Scotland; 2 Gloucestershire Hospitals NHS Foundation Trust, Cheltenham, GL53 7AN UK; 3 The Royal College of Ophthalmologists’ National Ophthalmology Audit, London, UK; 4 Guy’s and St. Thomas’ NHS Foundation Trust, London, UK; 5 Sunderland Eye Infirmary, Sunderland, UK; 6 Bioscience Institute, Newcastle University, Newcastle Upon Tyne, UK; 7 Moorfields Eye Hospital City Road, EC1V 2PD London, UK; 8 Royal Bournemouth Hospital Dorset, Bournemouth, UK; 9 James Paget University Hospital NHS Trust, Great Yarmouth, UK; 10 Royal Hallamshire Hospital, Sheffield, UK; 11 Brighton & Sussex University Hospitals Trust, Brighton, UK; 12 Martin University Hospital Slovakia, Martin, Slovakia; 13 Hull & East Yorkshire Eye Hospital, Hull, UK; 14 Maidstone & Tunbridge Wells NHS Trust, Royal Tunbridge Wells, UK; 15 Prince Charles Eye Unit King Edward VII Hospital, Windsor, UK; 16 Royal Victoria Infirmary, Newcastle upon Tyne, UK; 17 East Suffolk & North Essex NHS Foundation Trust, Essex, UK; 18 Manchester Royal Eye Hospital, Manchester, UK; 19 Musgrove Park Hospital Somerset NHS Trust, Taunton, UK; 20 Ninewells Hospital, Dundee, Scotland; 21 University Hospital North Midlands NHS Trust, Stoke-on-Trent, UK; 22 Sussex Eye Hospital, Brighton, UK; 23 Wolverhampton & Midland Counties Eye Infirmary, Wolverhampton, UK; 24 York Hospitals NHS Trust, York, UK; 25 Glangwili General Hospital, Carmarthen, UK

**Keywords:** Outcomes research, Risk factors

## Abstract

**Introduction:**

To identify variables associated with primary anatomical outcome following vitrectomy and internal tamponade for rhegmatogenous retinal detachment (RD).

**Methods:**

A retrospective analysis of prospectively collected data, using a database of RD treated with vitrectomy and internal tamponade. Collected data complied with the RCOphth Retinal Detachment Dataset. The main outcome measure was anatomical failure within six months of surgery.

**Results:**

There were 6377 vitrectomies. 869 eyes were excluded, either because no outcome was recorded, or inadequate follow up, leaving 5508 operations for analysis. 63.9% of patients were male, and the median age was 62. Primary anatomical failure occurred in 13.9%. On multivariate analysis, the following were associated with increased risk of failure: age <45, or >79, inferior retinal breaks, total detachment, one quadrant or greater inferior detachment, low density silicone oil, and presence of proliferative vitreoretinopathy. C_2_F_6_ tamponade, cryotherapy, and 25 G vitrectomy, were associated with reduced risk of failure. The area under the receiver operator curve was 71.7%. According to this model, 54.3% of RD are at low risk (<10%), 35.6% are at moderate risk (10–25%), and 10.1% are at high risk (>25%) of failure.

**Conclusions:**

Previous attempts to identify high risk RD have been limited by small numbers, the inclusion of both scleral buckling and vitrectomy, or by excluding some types of RD. This study examined outcomes in unselected RD, treated by vitrectomy. Identification of the variables associated with anatomical outcome after RD surgery enables accurate risk stratification, which is valuable for patient counselling and selection, and for future clinical trials.

## Abstract 2

**Learning Objectives:**

Upon completion of this activity, participants will:

1. Assess variables associated with primary anatomical outcome (anatomical failure within 6 months of surgery) after vitrectomy and internal tamponade for rhegmatogenous retinal detachment, based on a retrospective analysis of prospectively collected data
2. Evaluate risk stratification using a multivariate logistic regression model incorporating variables associated with anatomical failure within 6 months of rhegmatogenous retinal detachment surgery, based on a retrospective analysis of prospectively collected data
3. Determine the clinical implications of variables associated with primary anatomical outcome (anatomical failure within 6 months of surgery) after vitrectomy and internal tamponade for rhegmatogenous retinal detachment, based on a retrospective analysis of prospectively collected data.

**Accreditation Statements:**

In support of improving patient care, this activity has been planned and implemented by Medscape, LLC and Springer Nature. Medscape, LLC is jointly accredited with commendation by the Accreditation Council for Continuing Medical Education (ACCME), the Accreditation Council for Pharmacy Education (ACPE), and the American Nurses Credentialing Center (ANCC), to provide continuing education for the healthcare team.

Medscape, LLC designates this Journal-based CME activity for a maximum of 1.0 *AMA PRA Category 1 Credit(s)*™. Physicians should claim only the credit commensurate with the extent of their participation in the activity.

To participate in this journal CME activity: (1) review the learning objectives and author disclosures; (2) study the education content; (3) take the post-test with a 75% minimum passing score and complete the evaluation at www.medscape.org/journal/eye; (4) view/print certificate.

**Credit Hours:**

1.0

**Release date:**

**Expiration date:**

**Post-test link:** https://medscape.org/eye/posttest983835

**EDITOR:**

Sobha Sivaprasad, MD, Editor, *Eye*

**Authors/Editors disclosure information:**

David Yorston, FRCOphth, Gartnavel Hospital, Glasgow, Scotland. Paul H.J. Donachie, MSc, Gloucestershire Hospitals NHS Foundation Trust, Cheltenham, United Kingdom, The Royal College of Ophthalmologists, National Ophthalmology Database Audit, London, United Kingdom. D.A. Laidlaw, MD, FRCOphth, Guy’s and St. Thomas’ NHS Foundation Trust, London, United Kingdom. David H. Steel, MD, FRCOphth, Sunderland Eye Infirmary, Sunderland, United Kingdom, Bioscience Institute, Newcastle University, Newcastle Upon Tyne, United Kingdom. G.W. Aylward, MD, FRCOphth, Moorfields Eye Hospital City Road, London, United Kingdom. Tom H. Williamson, MD, FRCOphth, Guy’s and St. Thomas’ NHS Foundation Trust, London, United Kingdom

**Journal CME author disclosure information:**

Laurie Barclay has disclosed the following relevant financial relationships: formerly owned stocks in AbbVie Inc.

## Introduction

Some of the factors that affect anatomical success after retinal detachment (RD) surgery are relatively well understood, and grading systems have been produced that enable surgeons to predict the likelihood of anatomical re-attachment with a single operation [1–6]. However, some of these publications include both scleral buckling and vitrectomy [1, 4, 7, 8] The risk factors for failure are unlikely to be the same for these very different operations. Other reports have only included sub-sets of primary retinal detachments [3, 7], or have included re-operations [8]. Various different outcomes and definitions of anatomical success have been used [4, 5, 8]. Overall, the number of eyes included in each study has been small, with only one report including more than 1 000 eyes [8].

Previous authors have shown that the risk of primary anatomical failure may be increased by proliferative vitreoretinopathy (PVR) [3–6, 8], a greater extent of retinal detachment [3, 4, 9], foveal detachment [10] or total detachment [5, 8], the number of breaks in detached retina [5, 7], inferior breaks [5], the size of breaks [7], pseudophakia [3], hypotony or choroidal detachment [8], and the use of cryotherapy [7]. It is not surprising that there is little agreement between these studies, given the different inclusion criteria, interventions, and end-points, and the relatively small numbers of cases included.

In order to identify, and quantify, the variables associated with anatomical outcome of primary RD treated by pars plana vitrectomy and internal tamponade, we examined data from 5508 primary RD operations. In contrast to some of the other studies of risk factors, we included all primary rhegmatogenous RD, and used the outcome of primary anatomical failure, as this has been shown to be associated with worse functional outcomes [11, 12]

## Methods

The data for this analysis was recorded on the Britain & Eire Association of Vitreoretinal Surgeons (BEAVRS)/Euretina RD audit database which is compliant with the Royal College of Ophthalmologists national RD dataset [13]. The BEAVRS/Euretina database is an online web application for the collection, and analysis of anonymised vitreoretinal surgical data. Data is entered prospectively, immediately following surgery, and again when follow up is complete, at least two months post-surgery. The anatomical details of the retinal detachment are recorded using a drawing tool linked to diagnostic codes. This allows the accurate recording of RD extent, foveal attachment, retinal break location and type (U-tear, atrophic break, dialysis, schisis RD), and the presence, severity and extent of PVR using the Retina Society grading system [14]. The database is an audit tool rather than an electronic patient record. It only collects data relating to primary anatomical outcome, and does not record the results of re-operation.

Eligible operations were primary RD operations treated with a vitrectomy and internal tamponade with a recorded outcome of either surgical failure or success. All operations were performed between June 2008 and May 2019 where all cases of surgery success had at least 8 weeks follow up. RD secondary to penetrating injury, severe contusion, vasoproliferative disorders, inflammatory eye disease, or paediatric RD were excluded.

Anatomical failure was defined as surgeon recorded re-detachment, or a record of repeat RD surgery. Operations with an oil tamponade, and at least 140 days follow-up, but no record of oil removal, were presumed to be anatomical failures. In eyes in which the oil remained in situ and had less than 140 days follow-up, the outcome was recorded as unknown.

The probability of failure was modelled using multivariable logistic regression. All covariates under consideration were investigated at the univariate level using χ^2^ tests. Any covariate with a *p* < 0.10 progressed to multivariable modelling where the full model was fitted, and backward selection employed. A *p* < 0.05 plus assessment of Akaike Information Criterion and the area under the receiver operating curve were used for final covariate selection.

Robust standard errors were calculated using bootstrapping with 500 replications and clustering of the individual consultant responsible for the patient care, where the operations performed by the consultant surgeon were considered as a separate cluster to the operations performed by a trainee surgeon under their supervision.

The covariates considered were the use of laser photocoagulation, cryotherapy, type of tamponade, vitrectomy gauge size and sub-retinal fluid drainage route during surgery, patient’s age and gender, lens status, presence of age-related macular degeneration, amblyopia, glaucoma and myopia, the number of breaks in the detached retina, number of breaks in the attached retina, the location of the lowest break in the detached retina, the largest break type, the number of superior clock hours detached, the number of inferior clocks hours detached, total RD, PVR grade, schisis RD, foveal attachment and the post-surgery posturing.

Sensitivity analysis included different grouping for the patient’s age, PVR grade and vitrectomy gauge size.

All analyses were conducted using STATA version 16, (StataCorp. 2019. Stata Statistical Software: Release 16. College Station, TX: StataCorp LLC.), and 95% confidence intervals for failure rates were calculated using the Fleiss quadratic continuity correction [15].

This study conformed to the UK’s Data Protection Act and the principles of the Declaration of Helsinki. No patient could be identified with any of the data contained in the database and a unique random alphanumeric code is used for internal identification. As the dataset is considered a service evaluation, no IRB approval and/or informed consent were needed according to UK guidelines.

## Results

Within the study period, there were 7205 operations performed for primary RD. Of these, 6377 (88.5%) were vitrectomy with internal tamponade. No outcome was recorded for 162 (2.5% of these). A further 707 (11.1%) had a successful outcome recorded, but less than eight weeks follow-up, so were excluded. This left a total of 5508 (86.4%) procedures for analysis.

4815 (87.4%) operations were performed by 56 consultant surgeons, and 693 (12.6%) operations by trainee surgeons under the supervision of the consultant surgeon. The median number of operations performed by consultant surgeons was 47 (range; 1–575).

The median age at surgery was 62 years (Interquartile range = 54–70). 63.8% of patients were male and 54% of the operations were in right eyes. The detachment extended over one quadrant in 1102 (20.0%) eyes two quadrants in 2738 (49.7%) eyes, three quadrants in in 969 (17.6%) eyes, and four quadrants in 699 (12.7%) eyes. Total RD was present in 386 (7.0%) eyes. The fovea was attached in 2452 (44.5%) eyes.

1635 (29.7%) eyes had previous cataract surgery. Of these only 45 were aphakic. Age-related macular degeneration was recorded in 54 (1.0%) eyes, amblyopia in 83 (1.5%) eyes, glaucoma in 76 (1.4%) eyes and high myopia (>6D myopia) in 288 (5.2%) eyes.

### Surgical procedures

Vitrectomy was combined with a scleral buckle in 123 (2.2%) operations and phacoemulsification in 262 (4.8%) operations. Laser photocoagulation was used for 977 (17.7%) operations and cryotherapy for 3296 (59.8%) operations. Both laser photocoagulation and cryotherapy were used for 1235 (22.4%) operations. Drainage of sub-retinal fluid was recorded as through the break for 3714 (67.4%) operations, as via a retinotomy for 895 (16.3%) operations, as cutdown, needle or laser drainage for 38 (0.7%) operations and was not recorded for 861 (15.6%) operations.

The ocular tamponade used was sulphur hexafluoride gas for 2524 (45.8%) operations, perfluoroethane gas for 1711 (31.1%) operations, perfluoropropane gas for 681 (12.4%) operations, air for 47 (0.9%) operations, low density silicone oil for 447 (8.1%) operations and high density silicone oil for 98 (1.8%) operations. In the eyes that had light silicone oil, 310 (5.6%) had 1,000mPas oil, 33 (0.6%) had 2000 mPas oil, and 104 (1.9%) had 5000 mPas oil. In those that received heavy oil, 75 (1.4%) had Densiron (1300 mPas), and 12 (0.2%) had Oxane HD (3300 mPas)

The use of a gas or oil tamponade varied between the surgeons. For 39 consultant surgeons with at least 20 operations performed under their care, the use of oil tamponade ranged from 1.4% to 26.9% of operations, where 2 consultants used an oil tamponade in <5% of their operations and 4 consultants used an oil tamponade in ≥20% of theirs.

The vitrectomy gauge was 20 g for 192 (3.5%) operations, 23 g for 3 324 (60.4%) operations, 25 g for 1799 (32.6%) operations, 27 g for 88 (1.6%) operations and not recorded for 105 (1.9%) operations.

### Primary RD surgery failure model

Primary RD surgery failure occurred in 767 (13.9%) operations.

At the univariate level, no association was found between the patient’s gender (*p* = 0.177), the presence of glaucoma (*p* = 0.254) or myopia (*p* = 0.713), sub-retinal fluid drainage route (*p* = 0.266) or post-surgery posturing (*p* = 0.640), (Table 1). These variables were therefore excluded from multi variate analysis.

**Table 1:** Univariate analysis of covariates considered for the primary RD surgery failure model.

Covariate, *n* (row %)	Success	Failure	Total	*p*-value
Overall	4741 (86.1)	767 (13.9)	5508	N/A
Patient age (years)				
<45	290 (80.6)	70 (19.4)	360	<0.001
45 to 64	2549 (88.7)	324 (11.3)	2873	
65 to 79	1642 (84.7)	297 (15.3)	1939	
≥80	260 (77.4)	76 (22.6)	336	
Patient gender				
Male	3008 (85.6)	506 (14.4)	3514	0.177
Female	1733 (86.9)	261 (13.1)	1994	
Lens status				
No previous cataract surgery	3151 (87.3)	460 (12.7)	3611	0.002
Previous cataract surgery	1374 (84.0)	261 (16.0)	1635	
Combined phaco vitrectomy	216 (82.4)	46 (17.6)	262	
Foveal status				
Attached	2210 (90.1)	242 (9.9)	2452	<0.001
Detached	2519 (82.8)	522 (17.2)	3041	
Not recorded	12 (80.0)	3 (20.0)	15	
Number of breaks in the attached retina				
None	3359 (84.8)	601 (15.2)	3960	0.001
1 break	660 (89.4)	78 (10.6)	738	
2 breaks	312 (87.6)	44 (12.4)	356	
3 breaks	201 (89.3)	24 (10.7)	225	
>3 breaks	209 (91.3)	20 (8.7)	229	
Number of breaks in the detached retina				
None	14 (82.4)	3 (17.6)	17	0.008
1 break	2147 (87.5)	306 (12.5)	2453	
2 breaks	1113 (85.2)	194 (14.8)	1307	
3 breaks	685 (86.9)	103 (13.1)	788	
>3 breaks	782 (82.9)	161 (17.1)	943	
Location of largest break				
9 – 3 O’clock	3547 (89.0)	438 (11.0)	3985	<0.001
4 or 8 O’clock	477 (82.7)	100 (17.3)	577	
5 – 7 O’clock	703 (75.7)	226 (24.3)	929	
No break found	14 (82.4)	3 (17.6)	17	
Superior clock hours detached				
<3 h	1558 (86.4)	245 (13.6)	1803	<0.001
3 to 5 h	2673 (88.8)	338 (11.2)	3011	
6 h	510 (73.5)	184 (26.5)	694	
Inferior clock hours detached				
<3 h	2997 (91.3)	284 (8.7)	3281	<0.001
3 to 5 h	1068 (82.7)	224 (17.3)	1292	
6 h	676 (72.3)	259 (27.7)	935	
Total RD				
No	4502 (87.9)	620 (12.1)	5122	<0.001
Yes	239 (61.9)	147 (38.1)	386	
Largest break type				
Not found	33 (71.7)	13 (28.3)	46	<0.001
U tear	4162 (86.9)	627 (13.1)	4789	
Round hole	330 (81.7)	74 (18.3)	404	
Dialysis	32 (88.9)	4 (11.1)	36	
Giant Retinal Tear	137 (83.0)	28 (17.0)	165	
Macular hole	3 (42.9)	4 (57.1)	7	
Outer leaf break	44 (72.1)	17 (27.9)	61	
Schisis RD				
Absent	4697 (86.2)	750 (13.8)	5447	0.002
Present	44 (72.1)	17 (27.9)	61	
PVR grade				
None, A or B	4456 (88.1)	602 (11.9)	5058	<0.001
> = C	285 (63.3)	165 (36.7)	450	
Laser photocoagulation used during surgery				
No	2929 (88.9)	367 (11.1)	3296	<0.001
Yes	1812 (81.9)	400 (18.1)	2212	
Cryotherapy used during surgery				
No	764 (78.2)	213 (21.8)	977	<0.001
Yes	3977 (87.8)	554 (12.2)	4531	
Tamponade used during surgery				
Sulphur hexafluoride gas	2268 (89.9)	256 (10.1)	2524	<0.001
Perfluoroethane gas	1538 (89.9)	173 (10.1)	1711	
Perfluoropropane gas	558 (81.9)	123 (18.1)	681	
Air	42 (89.4)	5 (10.6)	47	
Light oil	265 (59.3)	182 (40.7)	447	
Heavy oil	70 (71.4)	28 (28.6)	98	
Vitrectomy gauge used				
20 g	143 (74.5)	49 (25.5)	192	<0.001
23 g	2809 (84.5)	515 (15.5)	3324	
25 g	1618 (89.9)	181 (10.1)	1799	
27 g	78 (88.6)	10 (11.4)	88	
Not recorded	93 (88.6)	12 (11.4)	105	
Sub-retinal fluid drainage route				
Retinotomy	755 (84.4)	140 (15.6)	895	0.266
Through the break	3208 (86.4)	506 (13.6)	3,714	
None/not recorded/other	778 (86.5)	121 (13.5)	899	
Patient post-surgery posture position				
None	1149 (86.1)	186 (13.9)	1335	0.640
Prone	1756 (86.2)	281 (13.8)	2037	
Upright	1048 (85.1)	183 (14.9)	1231	
Other	788 (87.1)	117 (12.9)	905	
Age-related Macular Degeneration				
Absent	4704 (86.2)	750 (13.8)	5454	<0.001
Present	37 (68.5)	17 (31.5)	54	
Amblyopia				
Absent	4677 (86.2)	748 (13.8)	5425	0.017
Present	64 (77.1)	19 (22.9)	83	
Glaucoma				
Absent	4679 (86.1)	753 (13.9)	5432	0.254
Present	62 (81.6)	14 (18.4)	76	
High Myopia				
Absent	4491 (86.0)	729 (14.0)	5220	0.713
Present	250 (86.8)	38 (13.2)	288	

**Fig. 1:**
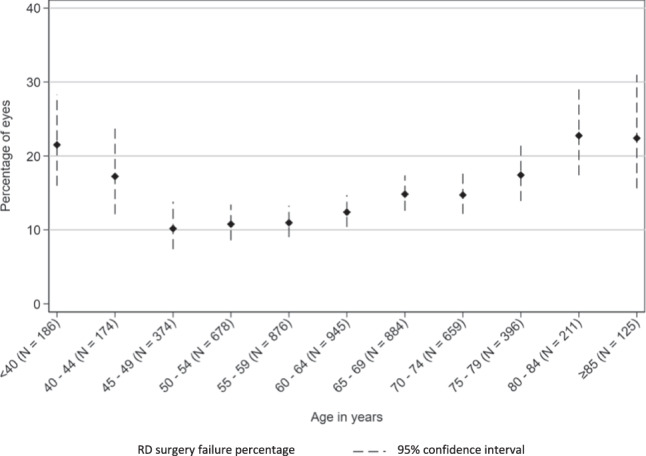
Primary RD surgery failure rates for 5-year age bandings. *N* = 5508 primary RRD operations performed under the care of 56 consultant surgeons. These data are unadjusted for case complexity.

**Fig. 2:**
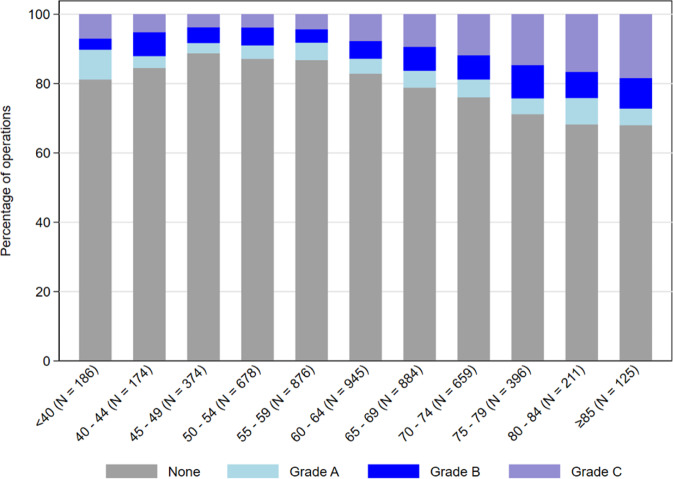
The percentage of primary RD operations with each grade of PVR in 5-year age bandings. *N* = 5508 operations performed under the care of 56 consultant surgeons.

The risk of failure appears to be linked to age (Fig. 1) with the two highest risk age groups being patients either under 45 years old, or 80 years or older. The risk of PVR grade C or worse at presentation was also higher in older patients (Fig. 2).

The final best fitting multivariable model for factors associated with outcome included the patient’s age, the location of the largest break in the detached retina, the number of inferior clock hours detached, total RD, PVR grade, use of cryotherapy, type of tamponade and vitrectomy gauge size, (Table 2). This final best fitting model did not include foveal attachment, the number of breaks in the detached retina, the number of breaks in the attached retina, the largest break type, the number of superior clock hours detached, lens status, schisis RD, the use of laser photocoagulation, or the presence of age-related macular degeneration or amblyopia. The final model had an AUC of 71.7%, and the Pearson Chi-squared goodness of fit test produced a *p*-value of 0.484. Different categorisations of the PVR type, vitrectomy gauge size and the patient’s age at surgery did not improve the model fit.

**Table 2:** Primary RD surgery failure model estimates.

Covariate	Odds ratio	Coefficient	*p*-value	95% CI for the odds ratio
Constant	0.120	−1.611	<0.001	0.088 to 0.452
Patient age (years)				
45 to 64	Ref	Ref	N/A	N/A
65 to 79	1.266	0.236	0.005	1.076 to 1.490
≥80	1.645	0.498	0.001	1.221 to 2.218
<45	1.583	0.459	0.004	1.154 to 2.171
Location of lowest break				
9–3 O’clock	Ref	Ref	N/A	N/A
4 or 8 O’clock	1.533	0.428	0.002	1.171 to 2.008
5–7 O’clock	1.835	0.607	<0.001	1.505 to 2.237
No break found	1.966	0.676	0.242	0.634 to 6.100
Inferior clock hours detached				
<3 h	Ref	Ref	N/A	N/A
3 to 5 h	1.554	0.441	<0.001	1.259 to 1.918
6 h	1.545	0.435	0.005	1.143 to 2.089
**Total RD**				
No	Ref	Ref	N/A	N/A
Yes	1.941	0.663	<0.001	1.411 to 2.668
PVR grade				
None, A or B	Ref	Ref	N/A	N/A
C	1.247	0.220	<0.001	1.133 to 1.372
Cryotherapy used during surgery				
No	Ref	Ref	Ref	Ref
Yes	0.657	−0.420	0.011	0.476 to 0.908
Tamponade used during surgery				
Sulphur hexafluoride gas	Ref	Ref	Ref	Ref
Perfluoroethane gas	0.659	−0.417	0.006	0.490 to 0.887
Perfluoropropane gas	0.901	−0.104	0.473	0.678 to 1.198
Air	0.853	−0.159	0.752	0.319 to 2.284
Light oil	1.954	0.670	<0.001	1.361 to 2.805
Heavy oil	1.031	0.030	0.931	0.522 to 2.037
Vitrectomy gauge used				
20 g	Ref	Ref	Ref	Ref
23 g	0.665	−0.408	0.258	0.327 to 1.349
25 g	0.413	−0.885	0.014	0.204 to 0.834
27 g	0.495	−0.703	0.132	0.198 to 1.127
Not recorded	0.478	−0.738	0.123	0.187 to 1.223

The lowest risk of failure identified from the model is 3.4% for a patient aged between 45 and 64 years old in whom the lowest break is above the horizontal midline, <3 h of inferior clock hours detached, no total RD and no PVR treated with a 25 g vitrectomy, cryotherapy and perfluoroethane gas. In contrast, an 82 year old patient, with a total retinal detachment, a break at 6 o’clock and grade C PVR, treated by 23 G vitrectomy, laser retinopexy, and silicone oil, has a predicted failure risk of 74.5%.

Some of the factors identified are not modifiable as the patient’s age, PVR type, extent of the detachment, and the location of retinal breaks will be determined prior to presentation. However, the use of cryotherapy, vitrectomy gauge size and ocular tamponade are potentially modifiable. It is probable that the presence of non-modifiable variables influences the surgeon’s choice of modifiable factors, for example approximately 39% of operation that used any oil tamponade were in eyes with PVR C. For gas and air tamponade, >80% of operations were combined with cryotherapy, while 64.2% of light oil and 60.2% of heavy oil operations were combined with cryotherapy (Table 3).

**Table 3:** Tamponade use for the primary RD surgery failure risk factor model covariates.

Covariate	SF6 gas	C2F6 gas	C3F8 gas	Air	Light oil	Heavy oil	Overall
Number of operations	2524	1711	681	47	447	98	5508
Patient age (years)							
45 to 64	1399 (55.4)	910 (53.2)	328 (48.2)	18 (38.3)	182 (40.7)	36 (36.7)	2873 (52.2)
65 to 79	886 (35.1)	582 (34.0)	249 (36.6)	12 (25.5)	165 (36.9)	45 (45.9)	1939 (35.2)
≥80	114 (4.5)	107 (6.3)	53 (7.8)	2 (4.3)	50 (11.2)	10 (10.2)	336 (6.1)
<45	125 (5.0)	112 (6.5)	51 (7.5)	15 (31.9)	50 (11.2)	7 (7.1)	360 (6.5)
Location of largest break							
9–3 O’clock	2306 (91.4)	1110 (64.9)	266 (39.1)	29 (61.7)	253 (56.6)	21 (21.4)	3985 (72.3)
4 or 8 O’clock	128 (5.1)	257 (15.0)	119 (17.5)	8 (17.0)	54 (12.1)	11 (11.2)	577 (10.5)
5–7 O’clock	88 (3.5)	338 (19.8)	290 (42.6)	10 (21.3)	137 (30.6)	66 (67.3)	929 (16.9)
No break found	2 (<0.1)	6 (0.4)	6 (0.9)	0 (0.0)	3 (0.7)	0 (0.0)	17 (0.3)
Inferior clock hours detached							
<3 h	1961 (77.7)	979 (57.2)	207 (30.4)	28 (59.6)	99 (22.1)	7 (7.1)	3281 (59.6)
3–5 h	357 (14.1)	492 (28.8)	266 (39.1)	16 (34.0)	112 (25.1)	49 (50.0)	1292 (23.5)
6 h	206 (8.2)	240 (14.0)	208 (30.5)	3 (6.4)	236 (52.8)	42 (42.9)	935 (17.0)
Total RD							
No	2463 (97.6)	1630 (95.3)	594 (87.2)	47 (100.0)	297 (66.4)	91 (92.9)	5122 (93.0)
Yes	61 (2.4)	81 (4.7)	87 (12.8)	0 (0.0)	150 (33.6)	7 (7.1)	386 (7.0)
PVR grade							
None, A or B	2483 (98.4)	1600 (93.5)	597 (87.7)	44 (93.6)	274 (61.3)	60 (61.2)	5058 (91.8)
C	41 (1.6)	111 (6.5)	84 (12.3)	3 (6.4)	173 (38.7)	38 (38.8)	450 (8.2)
Cryotherapy used during surgery							
No	372 (14.7)	315 (18.4)	89 (13.1)	2 (4.3)	160 (35.8)	39 (39.8)	977 (17.7)
Yes	2152 (85.3)	1396 (81.6)	592 (86.9)	45 (95.7)	287 (64.2)	59 (60.2)	4531 (82.3)
Vitrectomy gauge used							
20 g	97 (3.8)	30 (1.8)	23 (3.4)	0 (0.0)	32 (7.2)	10 (10.2)	192 (3.5)
23 g	1298 (51.4)	1161 (67.9)	476 (69.9)	8 (17.0)	323 (72.3)	58 (59.2)	3324 (60.3)
25 g	1072 (42.5)	452 (26.4)	160 (23.5)	5 (10.6)	83 (18.6)	27 (27.6)	1799 (32.7)
27 g	27 (1.1)	49 (2.9)	3 (0.4)	1 (2.1)	5 (1.1)	3 (3.1)	88 (1.6)
Not recorded	30 (1.2)	19 (1.1)	19 (2.8)	33 (70.2)	4 (0.9)	0 (0.0)	105 (1.9)

SF6 = Sulphur hexafluoride gas.

C2F6 = Perfluoroethane gas.

C3F8 = Perfluoropropane gas.

## Discussion

This study shows that the main variables associated with primary RD surgery outcome include the patient’s age, the location of the largest break in the detached retina, the number of inferior clock hours detached, total RD, PVR grade, use of cryotherapy, type of tamponade and vitrectomy gauge size.

The worse prognosis in elderly patients could be related to difficulty maintaining an effective post-operative posture. In addition, older patients were more likely to have PVR C at presentation (Fig. 2), which may imply an increased risk of developing PVR post-operatively compared to younger patients. Previous studies of risk factors have not found that older age increases the risk of failure [3–5, 7, 8], however, RRD is relatively uncommon in patients over 80. Only 6% of patients in this study were aged 80 or older. A report from Japan found no difference in the anatomical outcomes of vitrectomy for retinal detachment in elderly and young patients, however, their definition of elderly was >70 years [16]. In Israel, a study of vitrectomy outcomes in patients aged 85 or older found primary anatomical failure occurred in 45% [17]. A recent report from the US found that older patients were more likely to have complex RD, and that this led to lower single operation success rates [18]. In view of demographic trends, it is likely that RD surgery in patients over 80 will become more common, and surgeons should be aware of the increased risk of anatomical failure in this age group.

Other studies have noted a relatively low primary anatomical success rate for younger adults [19, 20]. Patients under 45 years old who have a vitrectomy for RRD may be more likely to have retinal detachments linked to inherited disorders, such as Stickler’s or Marfan’s syndrome, which may explain the worse prognosis for younger patients.

The observed failure rates for operations where sulphur hexafluoride (SF_6_) or perfluoroethane (C_2_F_6_) were used were both 10.1%. Perfluoroethane was used in more complex detachments, and on complexity based multivariate modelling significantly lower failure rates were found in patients in whom C_2_F_6_ was employed than in those in whom SF_6_ was used. To illustrate this point: C_2_F_6_ was used more often than SF_6_ in eyes which had the following characteristics associated with failure: PVR grade C (C_2_F_6_ 6.5% vs SF_6_ 1.6%), Breaks between 5 & 7 o’clock (19.8% vs 3.5%) and greater than 3 inferior clock hours of retinal detachment (42.8% vs 22.3%). Perfluoropropane (C_3_F_8_) had a higher rate of failure (18.1%), but was used in more complex RD. In multivariate analysis, the success rate of C_3_F_8_ was not significantly different to SF_6_ or C_2_F_6_.

The failure model did not find heavy oil to be associated with anatomical failure, but did show that light oil was linked to a higher risk of re-operation. Failure rates for operations where any oil tamponade is used can be affected by missing data for the removal of the oil tamponade, patients not returning or declining to have the oil removed, and the more frequent use in certain non-modifiable risk factors. There is considerable variation in the use of low density silicone oil as a primary tamponade. Given the high probability that use of low density oil is associated with an increased risk of re-detachment, we recommend avoiding the use of oil in situations where it is not medically indicated (e.g. wanting to fly).

We found that 25 G vitrectomy had a higher anatomical success rate than 20 G. However other authors have not noted any difference [21–23]. We believe our findings should be treated with caution, as only 192 (3.5%) of vitrectomies were 20 G in our series. Although success rates were higher for 25 G than 23 G, this was not significant in multivariate analysis, which suggests that 25 G may have been used in less complex RD with a better prognosis. Other authors have not found any difference in outcomes with 23 G vs. 25 G PPV [24]

Retinopexy with cryotherapy was associated with a reduced risk of failure. Cryotherapy, particularly in horseshoe tears, has been considered to increase the risk of PVR [25]. In the SPR study, cryopexy was associated with an increased risk of failure [7]. However, clinical trials, and some recent studies, have not shown any link between cryotherapy and re-detachment [10, 26, 27]. Our data confirms that, when used appropriately, cryotherapy is safe and effective.

In contrast to some other models [7], we did not find the number of retinal breaks to be associated with an increased risk of re-detachment. In our model, the location of the lowest break was more important than the number, as has been previously noted [5], although not all studies have found inferior breaks to increase the risk of failure [28]. A higher number of breaks increases the probability that at least one of them will be inferior, and this may explain the different findings in the SPR study [7].

Very few eyes (2.2%) in this series had a combined PPV and scleral buckle. Some previous studies have suggested that PPV alone achieves acceptable success rates for RD caused by inferior breaks [29–32]. However, this is not universally accepted [33]. Recently, a large series from the US found that PPV combined with an encircling buckle had a higher primary success rate than PPV alone, in phakic eyes [34]. As in the VIPER trial [35], the difference was not significant in pseudophakic eyes. In US series, the success rate for RD with a break between 5 and 7 o’clock, treated by PPV alone, was 76.8%, which is similar to the 75.7% we found. Encircling buckles are associated with myopic shift and an increased risk of anterior segment ischaemia, but our data confirms that there is room for improvement in the anatomical outcome of inferior break RD treated by PPV alone.

Prior cataract surgery carried an increased risk of failure on univariate modelling with a failure rate of 12.7% in phakic eyes and 16% in eyes that were pseudophakic at presentation (*p* < 0.002). However, pseudophakia was not linked to failure in our multi variate model, unlike in some other models [3]. We have previously shown that pseudophakic RD are more likely to have PVR C, inferior breaks, greater extent, and occur in older people than phakic RD [36]. Each of these factors was identified in our model as being linked to anatomical failure. In this study, PVR C was present in 9.6% of pseudophakic eyes, and 6.9% of phakic eyes (*p* = 0.0002). Inferior breaks between 5 and 7 o’clock were found in 21.3% of pseudophakic eyes, and 13.7% of phakic eyes (*p* < 0.0001). Total RD occurred in 10.3% of pseudophakic eyes, and 5.3% of phakic eyes (*p* < 0.0001) These data suggest that it may be the characteristics of the detachment associated with phakic status, rather than the phakic status itself, which promote success or failure.

Although some previous studies have indicated that a greater extent of detachment is associated with a higher risk of failure, this has not been a universal finding. There does not appear to be a linear relationship between the extent of the detachment and the risk of failure. However, total detachment is associated with an increased risk of failure, even when compared to detachments involving 11 clock hours of retina. A previous report identified total detachment as being associated with worse visual outcomes in successfully re-attached macula off retinal detachments [37]. A recent case control study showed that total RD had a significantly lower success rate than partial or sub-total RD, and worse visual outcomes [38]

In sub-total detachments, the inferior extent of the detachment appears to matter much more than the total number of clock hours detached. Although this may be partly due to inferior breaks, logistic regression showed that both inferior extent, and inferior breaks, were independent risk factors for failure.

Many other studies have shown that the presence of PVR C at presentation is associated with worse visual and anatomical outcomes [3–5, 8, 37]. We confirmed that PVR C at presentation is linked to a worse prognosis. This is supported by an increasing effect with greater extent of PVR C. The failure rate for eyes with PVR CP1 or CP2 was 31%, but for eyes with PVR CP5 or greater, it was 58%.

Although this is not the first attempt to develop a predictive model for anatomical outcomes of RD surgery, we believe it to be definitive. Previous models have been based on relatively small numbers of cases, have included both scleral buckles and vitrectomy, have excluded some retinal detachments, or have used varying definitions of success or failure. These limitations have led to uncertainty over which variables are truly important in determining the anatomical outcome of PPV for retinal detachment, producing a confusing variety of different conclusions.

The main strengths of this study are the large number of cases, inclusion of all primary rhegmatogenous retinal detachments, exclusion of eyes treated by scleral buckle alone, and a clear definition of primary anatomical success. The data was collected prospectively, by a large number of surgeons, in multiple sites and a novel feature is the use of a collaborative online audit tool, the BEAVRS/Euretina RD database. This study demonstrates that useful data may be collected from large numbers of patients, at minimal cost.

Limitations of this study include the absence of patient identifier to use patients as a cluster variable in the modelling. In another UK study it was found that bilateral RD occurred in 7.2% of patients within 10 years of the initial operation [39]. The same study also showed that the risk of second eye RRD was greatest in the first year, with more than 1:40 patients developing RRD in the other eye within 12 months of the initial RRD.

Although there was a minimum follow-up of 2 months, the follow-up period was not standardised. This was an unfunded real world complexity based audit, not a funded prospective study or trial. Pragmatically we set a minimum 2 months follow up, based on the real world practices of contributing surgeons, with the proviso that identified patients undergoing further surgery within six months would be reclassified as failure. In this study, 79% of failures occurred within two months of surgery. It is unlikely that many eyes were incorrectly classified as primary success. It is possible that a small number of failures were missed if the re-detachment occurred more than two months after primary surgery, and was treated at a different centre.

The vitrectomy gauge size was not recorded for 105 eyes and some of the model covariates include relatively small group sizes.

We cannot be certain that all contributors included all RD under their care. However, the characteristics of the RD included are comparable with those included in the Scottish RD survey [40]. The Scottish RD survey was a prospective study in which ascertainment of cases was ensured. This suggests that our sample is representative of RD in the UK.

Some uncommon potential risk factors, such as schisis RD, were not significant in the final model. This does not necessarily mean that they are not associated with a greater risk of failure, but rather that their rarity meant that they did not significantly influence the outcome of the entire population.

The database does not record the use of heavy liquids, as informal surveys of BEAVRS members have indicated that PFCL are rarely used by BEAVRS members in primary RD surgery, except in giant retinal tears.

The model should be applied with caution outside the UK. Retinal surgeons in the UK tend to have a minimalist approach to RD repair. For example, in a recent study of RD surgery in the US, out of 546 eyes treated by vitrectomy, 297 (54.4%) had a buckle as well, almost all of which were encircling [41]. In our study, only 5.4% of eyes had 360 degree laser retinopexy, but, in a recent case series from France, 43% of RD treated by vitrectomy had 360 degree laser retinopexy [42]. It is possible that the variables associated with outcomes may be affected by the predominant surgical practices.

We did not attempt to ascertain the cause of failure, as this is often difficult to determine.

Given that most risk factors are not modifiable, how does this help to improve the management of RD? Better risk stratification has numerous benefits. In our model, 54.3% of RD are at low risk (<10%) of failure, 35.6% are at moderate risk (10-25%), and 10.1% are at high risk (>25%). Better knowledge of the probability of failure enables us to give more precise information to our patients. Improved recognition of high and low risk RD means that we can identify cases that are suitable for trainees and fellows. Identifying the eyes that are most likely to require re-operation enables us to target additional interventions, such as combined PPV and scleral buckle, or long-acting tamponades, more precisely.

Interventions for RD vary widely between surgeons, and there is relatively little good quality evidence to support therapeutic decisions. Accurate risk stratification is an essential component of future randomised trials that will develop evidence to guide retinal surgeons.

### Summary

#### What is known about this topic

- Previous studies of anatomical success after retinal detachment surgery have produced a confusing plethora of different factors associated with anatomical outcome.
- Many of these studies have examined the results after both scleral buckle and vitrectomy, although the causes of failure in these very different operations are unlikely to be the same
- Some studies have only examined a sub-set of retinal detachments, excluding those with certain characteristics.

#### What this study adds

- We examined outcomes in a large cohort of unselected retinal detachments treated by vitrectomy and internal tamponade. This reflects real world practice in the UK.
- Multivariate logistic regression identified nine different factors that were associated with anatomical success or failure. These included the inferior extent of the detachment, which had not previously been shown to affect outcome.
- Using the regression model, we were able to stratify retinal detachments according to their risk of failure. This will help clinicians to give accurate prognoses to patients, and assist researchers in future clinical trials of interventions for retinal detachment.

## Data Availability

All the data are in the BEAVRS retinal detachment database, and can be accessed with the consent of the BEAVRS board and the BEAVRS retinal detachment study group.
